# Impacts of Interannual Ocean Circulation Variability on Japanese Eel Larval Migration in the Western North Pacific Ocean

**DOI:** 10.1371/journal.pone.0144423

**Published:** 2015-12-07

**Authors:** Yu-Lin Chang, Jinyu Sheng, Kyoko Ohashi, Mélanie Béguer-Pon, Yasumasa Miyazawa

**Affiliations:** 1 Institute of Marine Environmental Sciences and Technology, National Taiwan Normal University, Taipei, Taiwan; 2 Department of Oceanography, Dalhousie University, Halifax, Canada; 3 Application Laboratory, Japan Agency for Marine-Earth Science and Technology, Yokohama, Japan; University of Vigo, SPAIN

## Abstract

The Japanese eel larvae hatch near the West Mariana Ridge seamount chain and travel through the North Equatorial Current (NEC), the Kuroshio, and the Subtropical Countercurrent (STCC) region during their shoreward migration toward East Asia. The interannual variability of circulation over the subtropical and tropical regions of the western North Pacific Ocean is affected by the Philippines–Taiwan Oscillation (PTO). This study examines the effect of the PTO on the Japanese eel larval migration routes using a three-dimensional (3D) particle tracking method, including vertical and horizontal swimming behavior. The 3D circulation and hydrography used for particle tracking are from the ocean circulation reanalysis produced by the Japan Coastal Ocean Predictability Experiment 2 (JCOPE2). Our results demonstrate that bifurcation of the NEC and the strength and spatial variation of the Kuroshio affect the distribution and migration of eel larvae. During the positive phase of PTO, more virtual eels (“v-eels”) can enter the Kuroshio to reach the south coast of Japan and more v-eels reach the South China Sea through the Luzon Strait; the stronger and more offshore swing of the Kuroshio in the East China Sea leads to fewer eels entering the East China Sea and the onshore movement of the Kuroshio to the south of Japan brings the eels closer to the Japanese coast. Significant differences in eel migration routes and distributions regulated by ocean circulation in different PTO phases can also affect the otolith increment. The estimated otolith increment suggests that eel age tends to be underestimated after six months of simulation due to the cooler lower layer temperature. Underestimation is more significant in the positive PTO years due to the wide distribution in higher latitudes than in the negative PTO years.

## Introduction

Anguillid eels are widely distributed in the world’s oceans, with three main species in the Northern Hemisphere: European eel, American eel, and Japanese eel [1]. Eels are an important source of food for fish, mammals, turtles, and birds and serve as an important oceanographic indicator; therefore, they are of great interest in oceanography, meteorology, and biology [2]. The biology of Anguillid eels is, however, not well understood because there is little direct observational evidence on migration of Anguillid silver (maturing) eels to their spawning grounds, and return journeys of the larvae to growth areas in continental waters.

Eel recruitment has significantly declined in the past three decades [3]. The time-mean annual global eel catch from 1950 to 1986 was 2600 tons; however, it started declining in 1987, and by 2011, it was only 300 tons [4]. Several causes have been suggested for the decline in eel recruitment, including overfishing and habitat loss due to human activities [5]. Changes in ocean climate may significantly affect eel recruitment [6,7,8], e.g., glass (juvenile) eel recruitment indices correlate with the North Atlantic Oscillation and the Gulf Stream position [9].

**Fig 1:**
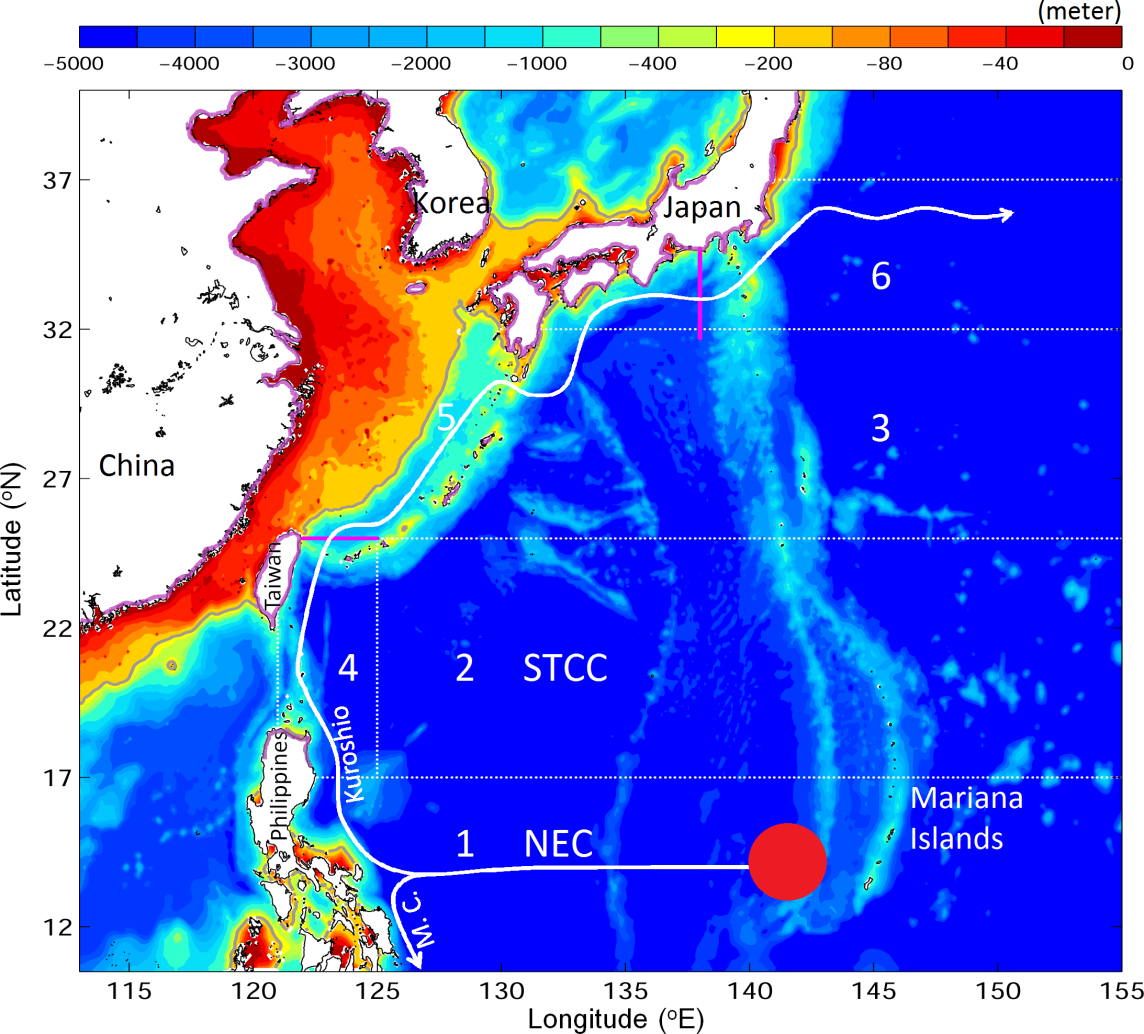
Major bathymetric features of the study area of the western North Pacific Ocean. Model results for regions marked by white dashed lines and associated numbers are presented in Fig 6. Results along sections marked by magenta lines are presented in Table 2. Solid white curves with arrows indicate major ocean currents. Abbreviations: North Equatorial Current (NEC); Subtropical Countercurrent (STCC); Mindanao Current (M.C.). The red spot marks the spawning ground. Purple contours along coastlines denote the arrival positions of eels after Tsukamoto [11].

**Fig 2:**
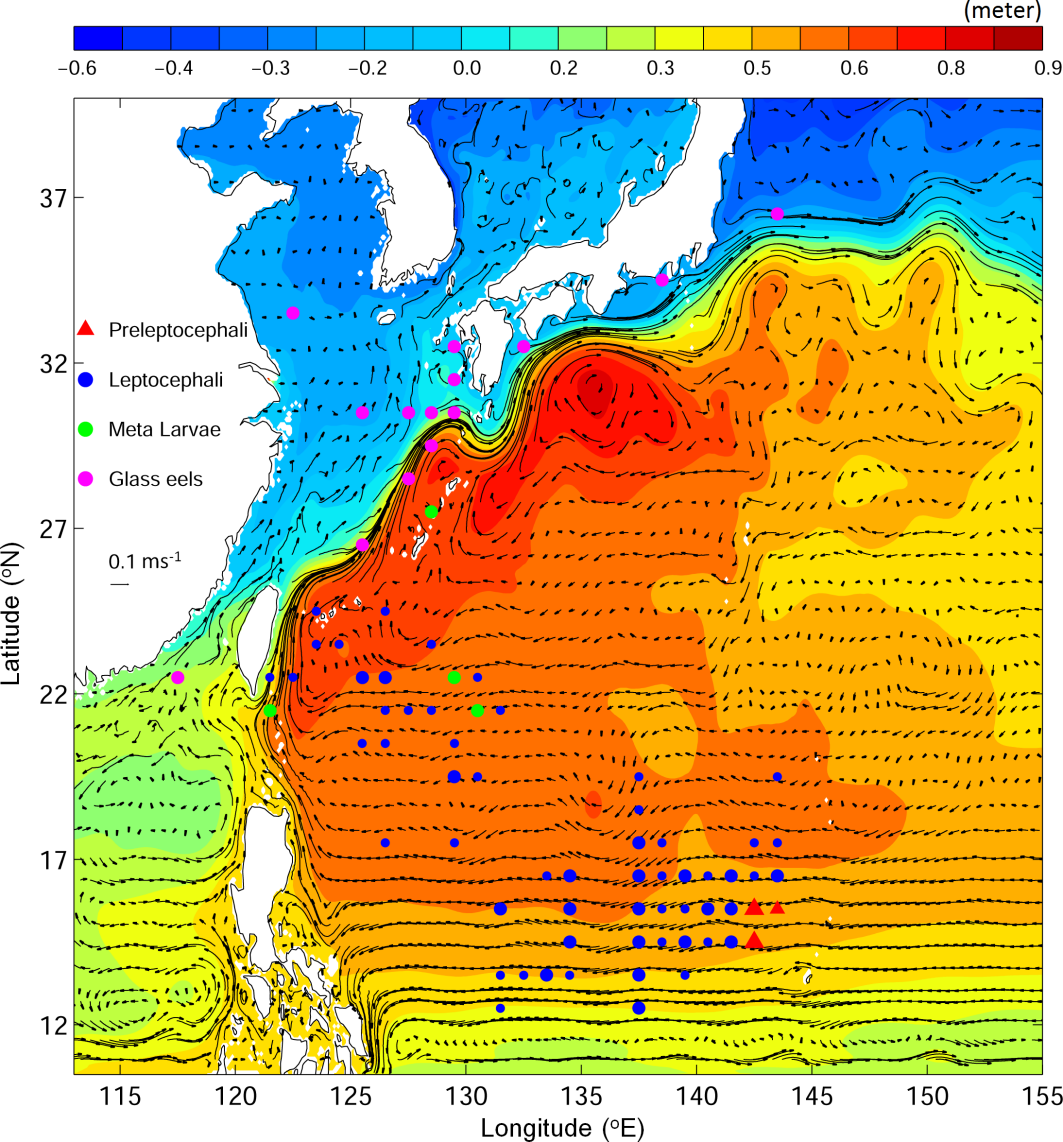
Vertically averaged ocean currents (vectors) in the top 300 m and time-mean sea surface height (m, image) over the 20-year period (1993–2012) calculated from the JCOPE2 reanalysis dataset. Colored dots denote observed eel larvae locations with different eel types represented by different colors (adapted from Shinoda et al. [14]).

The Japanese eel (*Anguilla japonica*) is a catadromous fish distributed in the western Pacific Ocean and listed as endangered on the IUCN red list [4]. Previous studies suggested that silver Japanese eels migrate seaward over a distance of thousands of kilometers to their spawning ground west of the Mariana Island in the Philippine Sea [10,11] (Fig 1). The newly born eel larvae depart from the spawning ground, carried primarily by ocean currents, toward their growth habitats in the fresh waters of East Asia [12,13] (Fig 1). Using 52-year observations (1956–2007), Shinoda et al. [14] examined the distributions of larval and juvenile Japanese eels in the western North Pacific (wNP). Preleptocephali (pre-larval eels) were found in the area near 142°E, 14°N to the west of the Mariana Islands from April to August (Fig 2, red triangles). Leptocephali (larval eels) were observed to be widely distributed, with their sizes increasing westward in the North Equatorial Current (NEC) to the east of Taiwan (Fig 2, blue circles). Metamorphosing larvae were detected to the east of Taiwan and the Okinawa Islands (Fig 2, green circles). Glass eels were found in the Kuroshio and the East China Sea in winter and early spring (Fig 2, magenta circles).

Though leptocephali are capable swimmers, their capacity for long-term sustained swimming is unknown [15]. Their swimming speeds are slow in comparison with typical ocean currents; thus, the movements and distributions of Japanese eels are strongly affected by ocean currents in their early life stages. Therefore, changes in environmental conditions should have a significant impact on eel dispersal and migration.

Otolith increments are widely used to study the life history of fish [16]. Ages estimated from otoliths have been used to reconstruct the spawning time of Japanese eels [11]. Fukuda et al. [17] indicated that otolith growth in *A*. *japonica* glass eels mainly depended on the ambient temperature rather than their feeding conditions. Otoliths generally grow in proportion to temperature. The otolith increment decreases with cooler temperature and ceases at temperatures lower than 10°C. Changes in environmental conditions under different climate phases may affect the water temperature, which may in turn influence the otolith increments and age estimates.

The effect of the El Nino/Southern Oscillation (ENSO) on Japanese eels has been examined previously [18,19,20,21]. Kimura et al. [20] proposed a possible link between the ENSO, the salinity front near NEC, and the eel catch based on historical data. Kim et al. [19] and Zenimoto et al. [21] suggested that the transport of particles carried by currents from the NEC to the Kuroshio is lowest in the El Nino years compared to the non-El Nino periods. Han et al. [18], however, detected no significant differences in the glass eel catch between the El Nino, La Nina, and normal years for the period 1972–2008. Tzeng et al. [22] concluded that the response of the eel catch to the El Nino is not significant based on the long-term (1967–2008) eel catch data and suggested that the Japanese eel recruitment may be influenced by multi-timescale climate variability.

**Fig 3:**
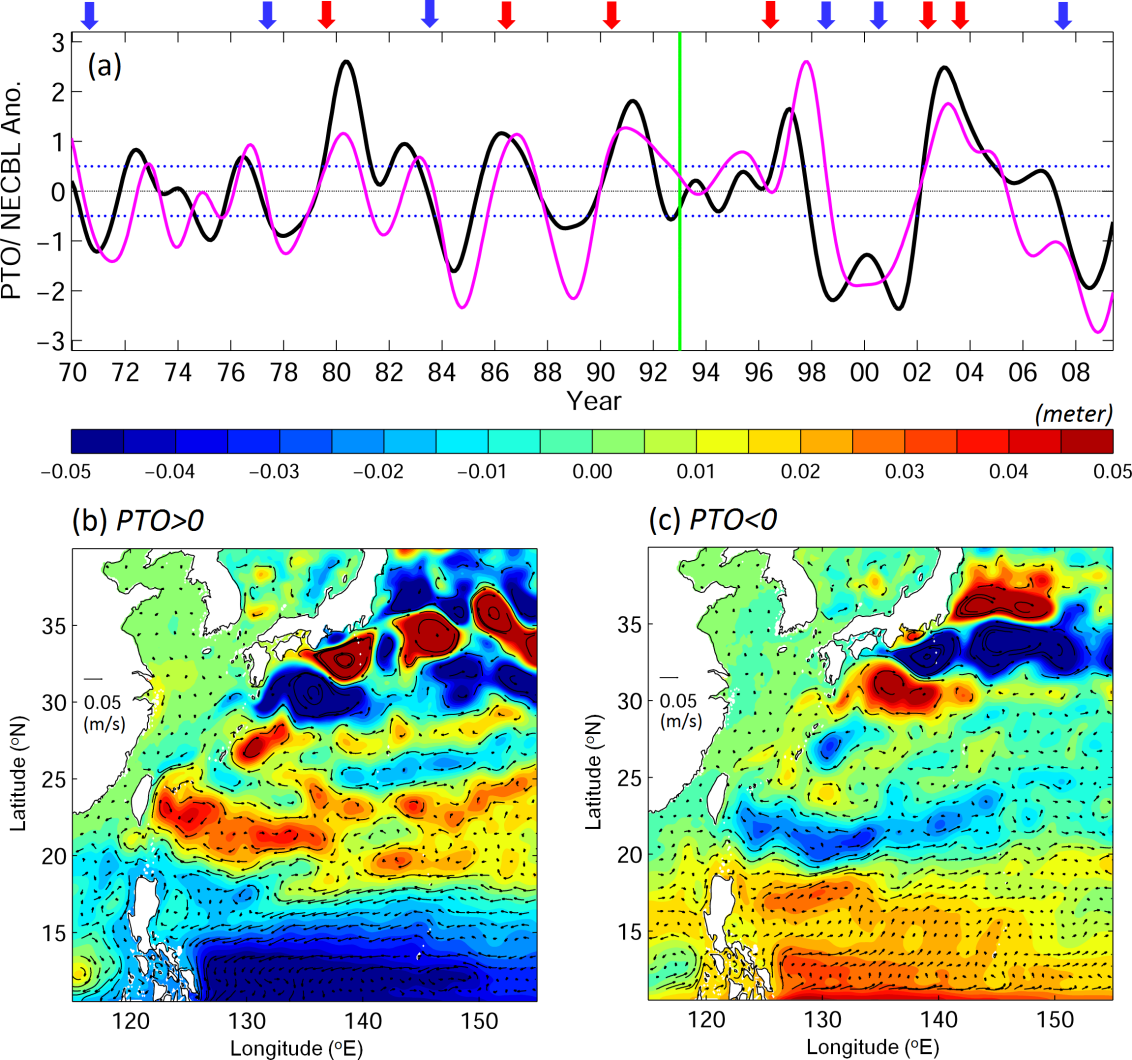
(a) Time series of PTO (black) and NEC bifurcation latitude (NECBL, magenta; Qiu and Chen, [44]) anomaly. Red and blue arrows mark the chosen years for positive and negative PTO, respectively. Lower panels show 20-year (1993–2012) composite surface current anomaly trajectories (ms^-1^) and sea surface height anomalies (m) for (b) the positive and (c) the negative PTO years calculated from the JCOPE2 reanalysis.

A newly developed climate index known as the Philippines–Taiwan Oscillation (PTO, Fig 3; [23]) has been successfully used to explain the interannual variability of subtropical and tropical circulation in wNP. The PTO represents the interannual oscillation of the oceanic thermocline to the east of the Philippines and Taiwan, forced by the corresponding oscillation in wind stress curls. In the positive PTO years, the thermocline rises to the east of the Philippines and deepens to the east of Taiwan. This thermocline seesaw results in a northward shift of the NEC, increased vertical shear of NEC/Subtropical Countercurrent (STCC) system, enhanced eddy activity in STCC region, strengthened Kuroshio transport off Taiwan, and larger Luzon Strait intrusion into the South China Sea (Fig 3; [23]). The region affected by the PTO covers wNP, including the NEC, the Kuroshio, and the STCC eddy region, where the eel larval migration routes are located.

This study examines the effect of changes in physical environmental conditions associated with the PTO on the Japanese eel larval migration over wNP. A three-dimensional (3D) particle tracking method is used to simulate the movement of virtual eels (hereafter v-eels) with swimming behavior carried by the 3D currents. We investigate the distribution and migration of v-eels under different climate scenarios, determine the factors affecting eel distributions, and assess the application of otolith increment using along-track temperature in two climate regimes. We also examine the sensitivity of simulated particle movements on the horizontal resolution of the ocean currents and the importance of horizontal swimming ability.

### Data and methods

An individual-based model is used in this study to simulate biological processes at the level of individuals or small groups of individuals in the population [24]. Ocean circulation reanalysis is used to drive the individual-based model to track movement of particles carried by ocean currents with inclusion of biological behaviors such as diel vertical migration and horizontal swimming.

### Ocean reanalysis

The 3D currents and hydrological fields used in particle tracking were extracted from the ocean circulation reanalysis of the Japan Coastal Ocean Predictability Experiment 2 (JCOPE2; [25]). JCOPE2 used a data-assimilated ocean model constructed from the Princeton Ocean Model (POM) with a generalized coordinate system [26]. The JCOPE2 model domain encompasses wNP (10.5–62°N, 108–180°E), with a horizontal resolution of 1/12° (8–9 km) and 46 vertical layers. The model external forcing includes wind stress and net heat/freshwater fluxes at the sea surface converted from six-hourly atmospheric reanalysis produced by the National Centers for Environmental Prediction/National Center for Atmospheric Research (NCEP/NCAR). Satellite and in situ temperature and salinity data were assimilated into the model. Daily JCOPE2 reanalysis fields cover the time period from January 1993 to the present. Details of the model have been described previously [25].

The European Centre for Medium-Range Weather Forecasts Ocean Reanalysis System 4 (ECMWF-ORAS4) dataset is also used to examine the sensitivity of particle movements to the spatial and temporal resolutions of the ocean circulation fields. ECMWF-ORAS4 was produced by an operational global ocean reanalysis system [27], with a horizontal resolution of 1° and 42 z-levels vertically. The ORAS4 dataset has a monthly interval and covers the period from 1958 to the present.

### Particle tracking scheme

#### Particle movement caused by ocean currents

This study uses the 3D particle tracking scheme used by Ohashi and Sheng [28] based on the fourth-order Runge–Kutta method [29]. The position of a particle is tracked from its position at time *t (* ${\vec{x}}^{t}$ *)* to a new position at time *t* + Δ*t (* ${\vec{x}}^{t+\Delta t}$ *)* based on

$${\vec{x}}^{t+\Delta t}={\vec{x}}^{t}+\int_{t}^{t+\Delta t}\vec{u}(\vec{x},t)dt+\vec{\delta }$$ (1)

where $\vec{u}$ is the 3D ocean current vector from the JCOPE2 (or ORAS4) reanalysis, and $\vec{\delta }$ represents the additional displacement during this time interval associated with a random walk, representing unresolved sub-grid turbulent flow and other local processes [30]. The same tracking scheme was used by Sheng et al. [31] to examine dispersion and retention in Lunenburg Bay, Canada.

#### Particle movement caused by active swimming

Two important biological behaviors of Japanese eels are considered in this study: diel vertical migration (DVM) and horizontal swimming. Eel larvae show DVM behavior, i.e., they remain in upper surface waters at night and dive to deeper waters to avoid predators during the daytime. Castonguay and McCleave [32] observed that *Anguilla* leptocephali of length 5.0–19.9 mm are present mostly at depths of 100–150 m by day and 50–100 m by night. Larger *Anguilla* larvae (≥20 mm) were found in deeper layers (125–275 m) during the day and mostly between 30 and 70 m at night. In this study, the age-dependent DVM of the v-eels is defined as follows:

$$z=\{\begin{array}{c} 50+0.75t\;\text{during the day}\\ 50\;\text{at night} \end{array}$$ (2)

where *z* represents the depth of the v-eel (m) and *t* is eel age (days). A 100- and 200-day old v-eel, for example, can dive to 125 and 200 m, respectively, during daytime. The linear relation with time described in Eq 2 represents the increase in diving depth with eel growth. Day and night times are determined by sunrise and sunset each day. Sunrise and sunset are set seasonally. Day length in spring and autumn is set to 12 hours (6 am to 6 pm) and is shortened to 10 hours (7 am to 5 pm) in winter (December–February). A longer day length of 14 hours (5 am to 7 pm) is set in summer (June–August).

This study of particle movement with age-dependent DVM differs from previous studies. Most previous studies were based on particle movement in specific vertical layers [6,33] or on DVM between two specific layers [34,35]. In this study, particle movements are 3D.

Horizontal swimming speeds of leptocephali were approximately 3.6 ± 2.7 cms^-1^ in laboratory experiments [36]. Wuenschel and Able [37] suggested that the short-term swimming speed of glass eel has a maximum value of 13 cms^-1^ and the long-term swimming speed is ~6 cms^-1^. In this study, the horizontal swimming speed of eel larvae is set to increase linearly with time:

$$\text{swimming speed}=\;0.06\;\text{t}\;\left(cm\;s^{-1}\right)$$ (3)

by assuming that the swimming speed of newborn eels is very small. The swimming direction of the v-eel is set to be the same as the local flow in the open ocean with water depths greater than 100 m. From Eq (3), v-eels which are 100- and 200-days old have swimming speeds of 6 and 12 cms^-1^ in the open ocean, respectively. A similar approach was used by Rypina et al. [34] to simulate American eel migrations. When the v-eels arrive over coastal and shelf waters with water depths shallower than 100 m, they are set to search for lower salinity and swim toward coastal fresher waters.

### Otolith increments

The wide range of temperatures in the study region can influence the otolith increment, leading to different estimates of eel ages. Based on an experimental study of glass eels [17], and assuming the same applies to leptocephalus stages, the number of otolith increments per day (*G*_*r*_) is set to:

$$G_{r}=\{\begin{array}{ll} \;0, & \;T<10{}^{\circ}C\\ (T-10)\times 0.09, & 10{}^{\circ}C\leq T\leq 21{}^{\circ}C\\ \;1, & \;T>21{}^{\circ}C \end{array}$$ (4)

where *T* is the water temperature. A similar formula was used previously by Zenimoto et al. [35] to calculate otolith increments for larval *A*. *anguilla* and *A*. *japonica*.

### Experimental design

Several numerical experiments are conducted to examine v-eel migration under different climate regimes. We follow Chang and Oey [23] and define positive PTO years as years in which the PTO index exceeds half a standard deviation (Fig 3). During the study period, the positive PTO years are 1996, 2002, and 2003 and the negative PTO years are 1998, 2000, and 2007. The release region and period of v-eels are chosen on the basis of the observed spawning ground and season. The spawning area of Japanese eels is located near the west of Mariana Islands [12]. The particles (v-eels) are released over the region 140 to 142°E and 13 to 15°N with a separation distance of 10 km (with 440 v-eels). Preleptocephali were observed in June [11]. Leptocephali estimated to be 10–40 days old were found in June and July [12].The initial release time is set from May 1 to July 31, and staggered by a time interval of 5 days during the three-month period. The Japanese eels were born in summer near the Mariana Islands, and were observed to reach East China Sea and south of Japan in winter and early spring [14]. The migration period estimated from observations is about six to eight months [14]. The duration of passive particles’ migration from the Mariana Islands to the area south of Japan has been estimated to be seven months [35]. The tracking duration is set to be eight months to track v-eels’ shoreward migration route.

The 3D circulation fields from the coarse-resolution ECMWF dataset are used in this study to check the sensitivity of the particle-tracking experiments to the spatial resolution of currents. In addition to the years selected above for different PTO phases, six more years are chosen for the composite (Fig 3A): 1979, 1986, and 1990 for the positive PTO phase and 1970, 1977, and 1983 for the negative PTO phase.

Six numerical experiments are conducted in this study (Table 1). In Exp01, v-eels are passively carried by the ocean currents. In Exp02, v-eels have swimming ability based on Eq (3). Exp03 tests the sensitivity of swimming speed and eel size after metamorphosis into glass eels. Exp04 is used to examine the slower swimming speed. Exp05 repeats the two-layer method used in former studies. Exp06 tests the effect of model resolution.

**Table 1:** Parameters used in the particle-tracking experiments.

Exp. Name	Ocean Model	Resolution	DVM	Swim Speed
Exp01	JCOPE2	1/12°	size-dependent, following Eq 2	None
Exp02	JCOPE2	1/12°	size-dependent, following Eq 2	size-dependent, following Eq 3
Exp03	JCOPE2	1/12°	size-dependent, following Eq 2	size-dependent, following Eq 3, but constant after day 150
Exp04	JCOPE2	1/12°	size dependent, following Eq 2	size-dependent, reduced parameter to 0.05 in Eq 3
Exp05	JCOPE2	1/12°	two-layer, day at 150 m, night at 50 m	size-dependent, following Eq 3
Exp06	ECMWF	1°	size-dependent, following Eq 2	size-dependent, following Eq 3

## Results

### Validation of JCOPE2 currents

**Fig 4:**
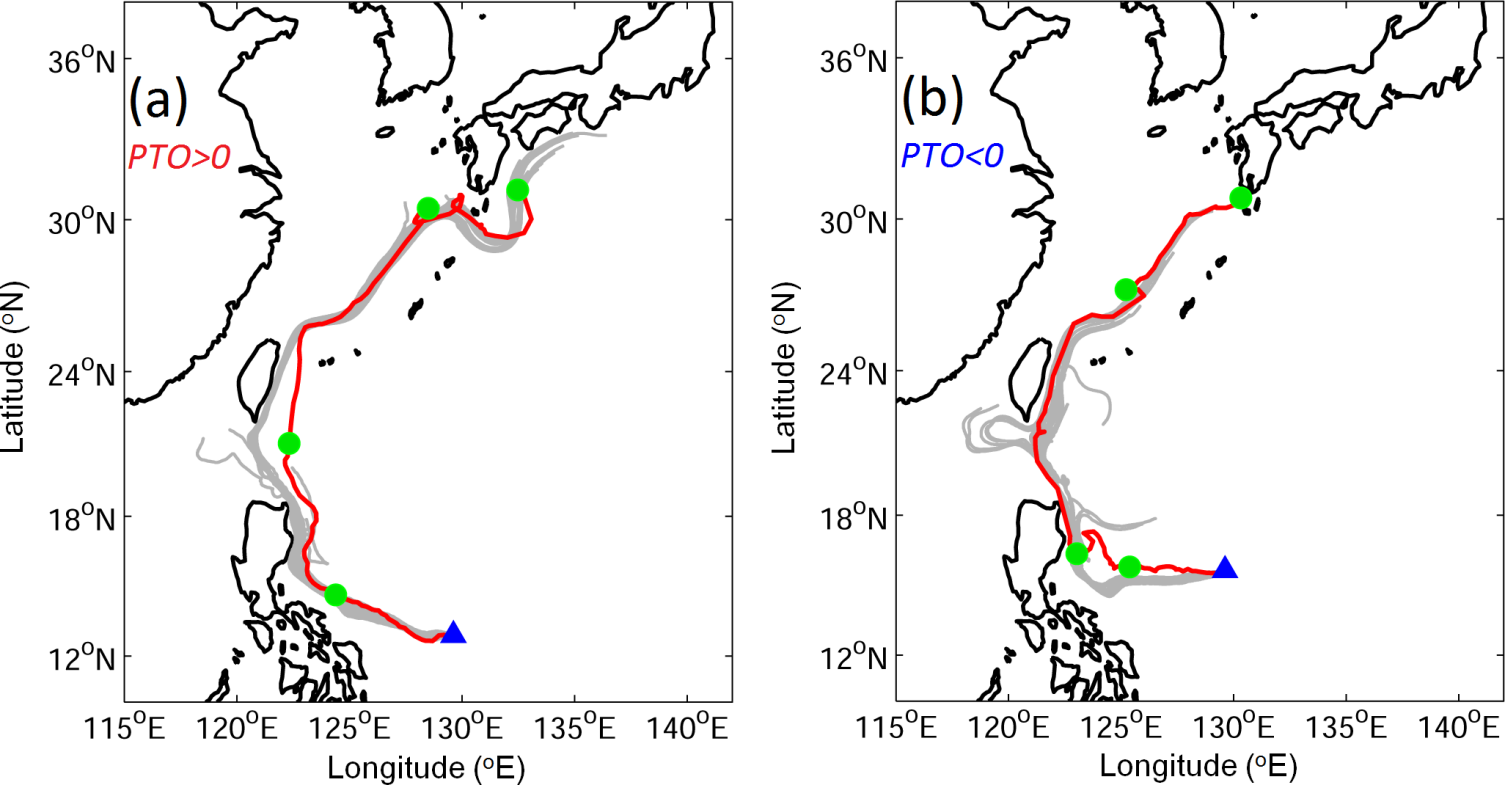
Observed (red) and simulated (gray) trajectories of surface drifters for (a) positive PTO and (b) negative PTO. Blue triangles represent the starting locations. Green circles mark the positions at day 30, 60, 90, and 120. Tracking period is 120 days.

To demonstrate the performance of particle movements using the JCOPE2 currents, the simulated trajectories of passive particles carried by the JCOPE2 currents are compared with the observed trajectories of two selected surface drifters in the positive and negative PTO years on November 24, 2003 and January 31, 2009, respectively. The drifter data were obtained from the Global Drifter Program (http://www.aoml.noaa.gov/phod/dac/index.php). The two drifters were deployed near 130°E at the surface. Passive particles are released over an area and on a day close to the observed drifter release location and time. Fig 4 demonstrates that the modeled trajectories of passive particles are in good agreement with the observed drifter trajectories. Simulated trajectories of passive particles also show the possible intrusion through the Luzon Strait (Fig 4). The observed and simulated drifters in the positive PTO year move faster than in the negative PTO year. The longer distance and faster travelling drifters suggests the stronger Kuroshio in the positive PTO year than in the negative PTO year.

**Fig 5:**
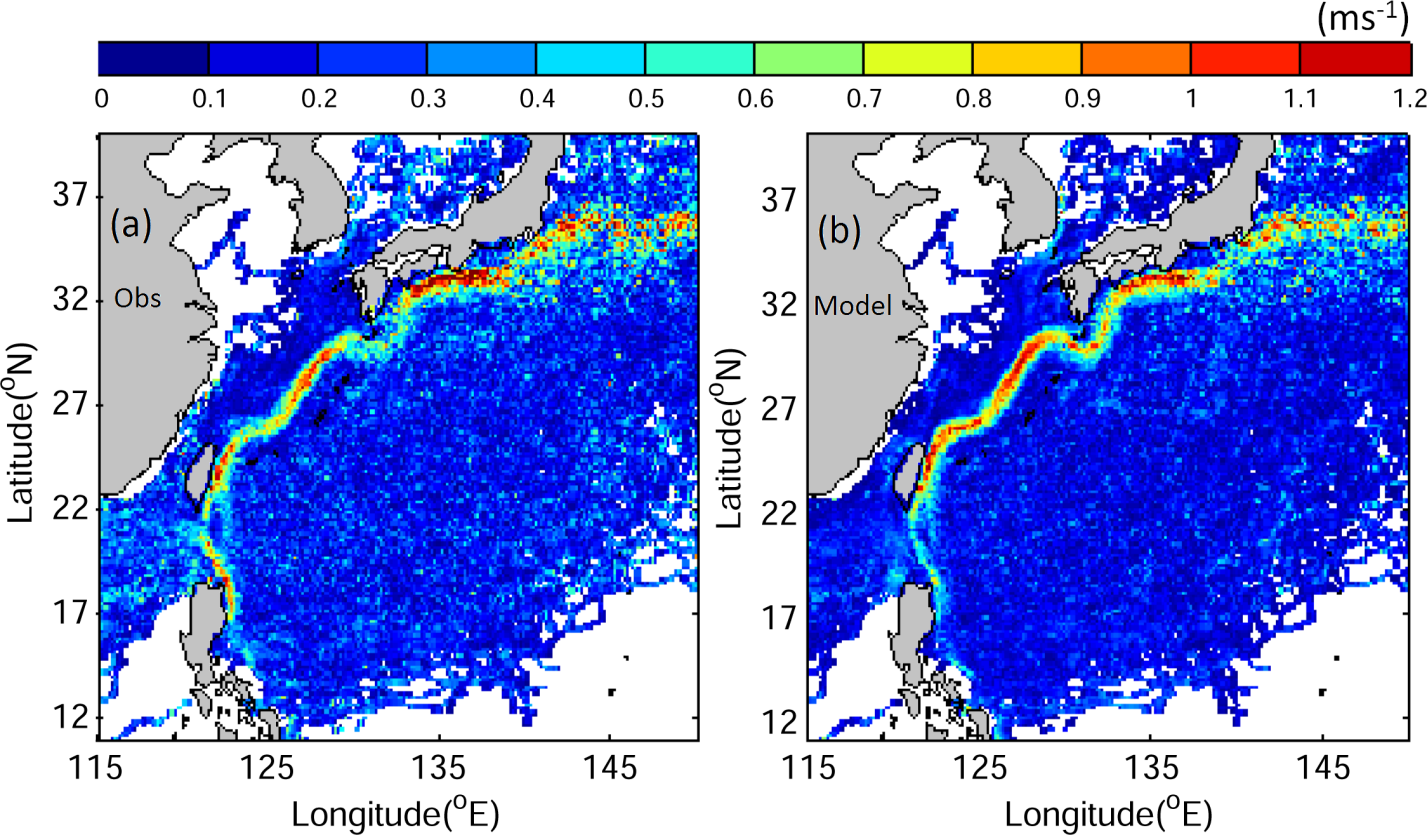
(a) Distributions of along-track speeds (ms^-1^) of surface drifters calculated from observed trajectories of 820 drifters and (b) simulated along-track speeds interpolated from model results for the same times and locations as the observations.

We next compare the model currents with the along-track speeds inferred from observed trajectories of 820 surface drifters deployed during the period 1993–2012. The model currents from JCOPE2 are interpolated to the same locations at the same times as the inferred along-track speeds of surface drifters for direct comparison. Fig 5 demonstrates that the JCOPE2 reanalysis reproduces the general patterns of along-track speeds of surface drifters well, with high along-track speeds in the path of the Kuroshio and much lower speeds over other regions. However, JCOPE2 overestimates the Kuroshio in the East China Sea and slightly underestimates the jet to the east of Luzon Island and to the south of Japan.

**Fig 6:**
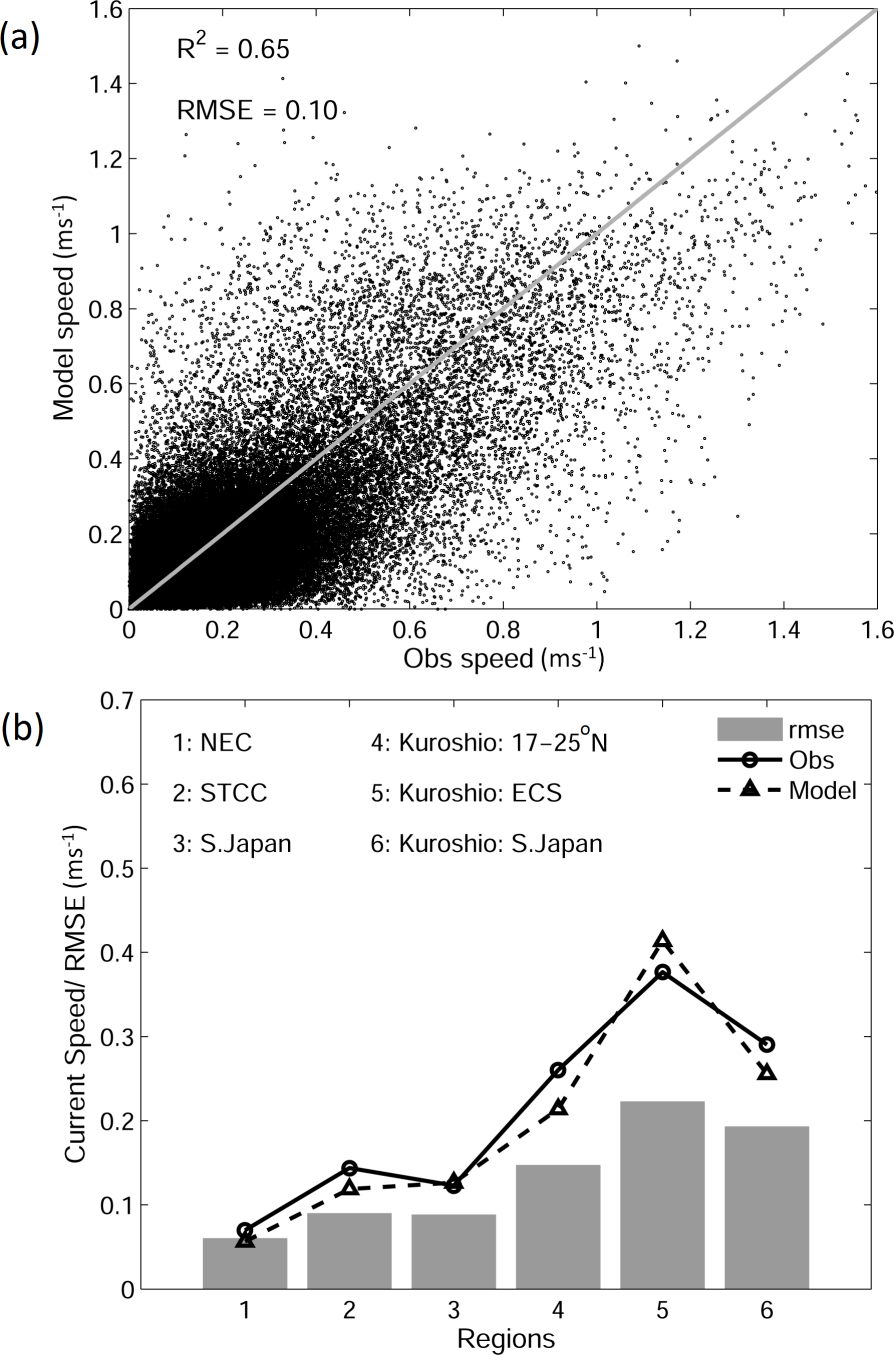
(a) Scatterplot of observed and simulated along-track speeds (ms^-1^) of surface drifters and (b) area-averaged values of observed and simulated speeds and RMSE between them for six sub-regions (marked in Fig 1).

The scatterplot of observed and simulated along-track speeds of surface drifters (Fig 6A) further demonstrates that the JCOPE2 reanalysis performs reasonably well in reproducing the observed along-track speeds of surface drifters, but with a certain degree of scatter. The r-squared value between observed and simulated along-track speeds is ~0.65, with a root-mean-square error (RMSE) between observed and simulated speeds of ~0.1 ms^-1^. Fig 6B shows area-averaged values of observed and simulated along-track speeds and RMSE values for six sub-regions marked in Fig 1. Overall, the JCOPE2 reanalysis slightly underestimates the along-track speeds in the NEC, STCC, upstream Kuroshio to the south of 25°N, and the Kuroshio off southern Japan and overestimates the Kuroshio in the East China Sea. RMSE is less than 0.1 ms^-1^ in the open ocean and approximately 0.15–0.20 ms^-1^ in the Kuroshio, which indicates that the JCOPE2 reanalysis reproduces the general circulation in the study region well, particularly the path and strength of the Kuroshio.

### Effect of circulation variability on v-eel distributions

**Fig 7:**
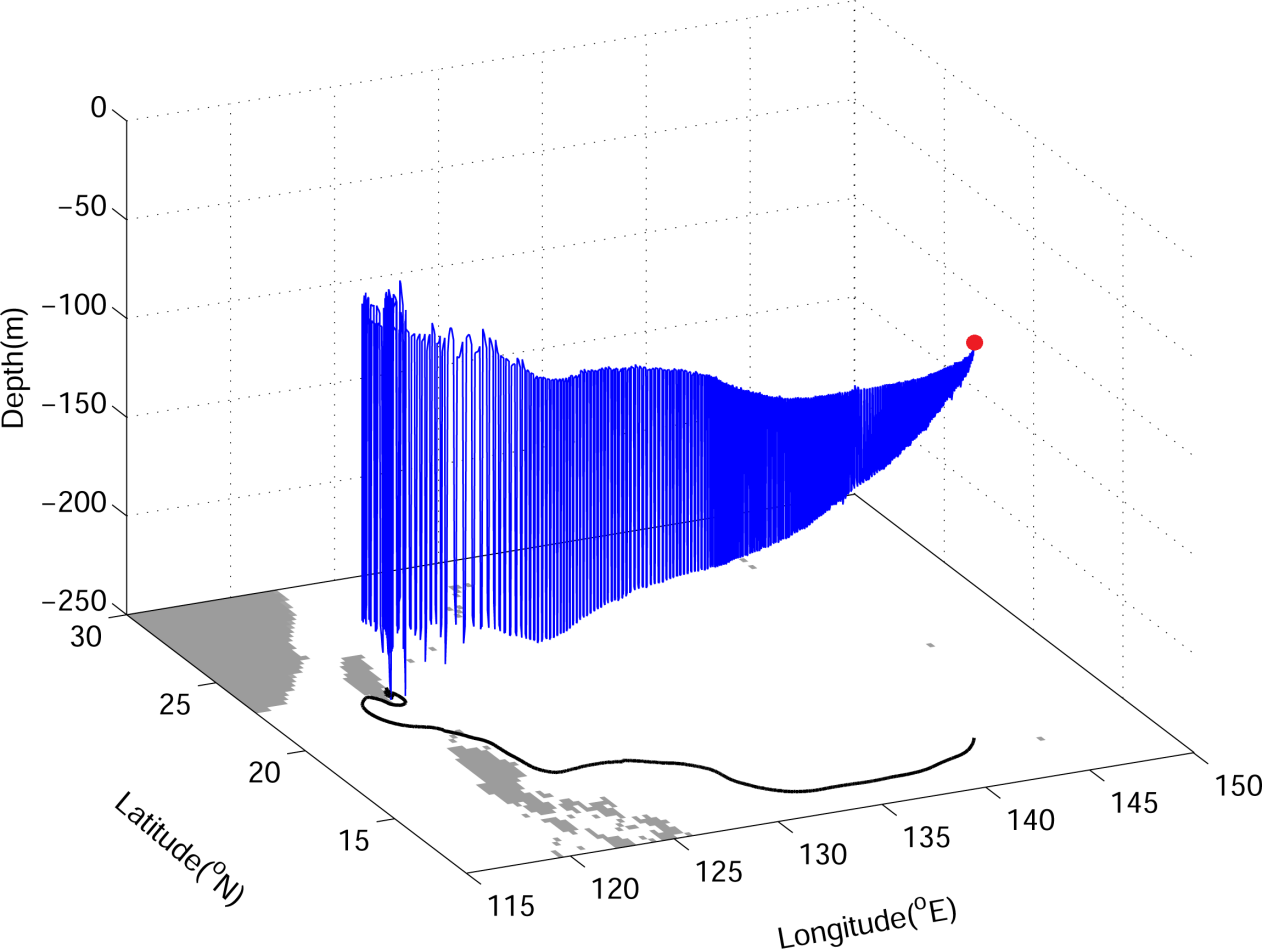
Example of a 3D simulated eel trajectory (blue) from Exp02 with active swimming behavior. The black curve is the horizontal projection; the red point marks the release position.

Fig 7 presents an example of the 3D trajectory of a v-eel released at 140°E and 14°N on May 1, 1996 for eight months of tracking in Exp02. This v-eel first drifts with the NEC, then joins the Kuroshio, and reaches the east coast of Taiwan at the end of tracking. The v-eel has stronger DVM with growth, according to Eq 2.

**Fig 8:**
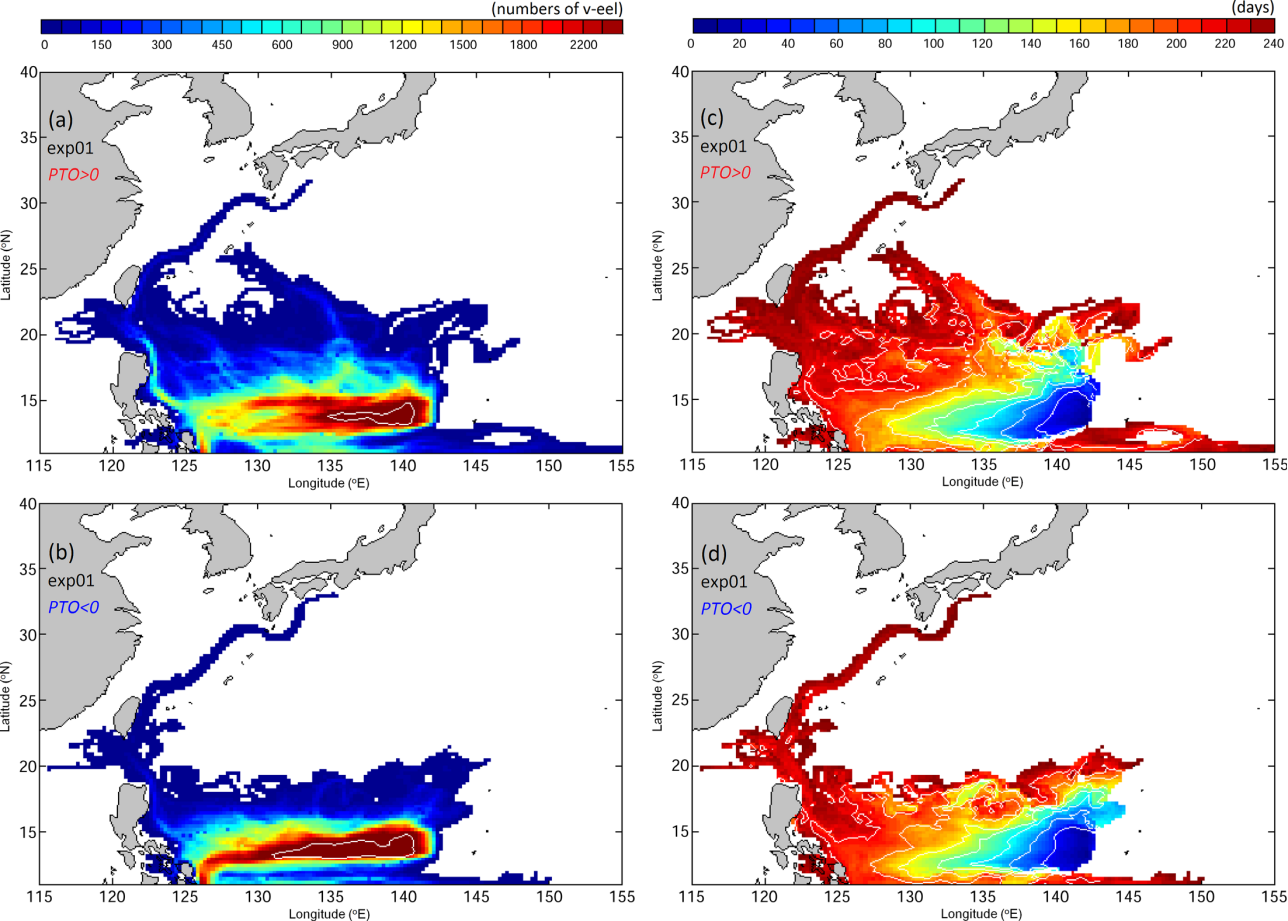
VF distributions (left, number of times) and age (right, days) from eight-month simulations of v-eels without horizontal active swimming behavior in Exp01 for positive (top) and negative (bottom) PTO phases.

To quantify the movements and distributions of v-eel trajectories, the depth-integrated particle trajectories are composited into a 2D field known as the visitation frequency (VF), which represents the frequency of particle arrivals in each grid cell. Fig 8 shows the VF distributions of v-eels without active swimming ability (passive) for eight months after release for the positive and negative PTO phases in Exp01. The v-eels in Exp01 are transported westward passively by the NEC; most remain in the NEC region at the end of tracking. Only a few v-eels reach the Kuroshio in eight months. The movements of passive v-eels are slower than the observed movements of eel larvae (Fig 8A and 8B and Fig 2). This is in contrast to the findings of Zenimoto el al. [35], which demonstrated that eel larvae can arrive at 30°N to the south of Japan in seven months using numerical tracking.

Age distributions of passive v-eels in Exp01 (Fig 8C and 8D) indicate the spreading of particles with time. Most v-eels carried passively by the NEC reach the region east of the Philippines in 210 days. The v-eels require more than 210 days to arrive in the Kuroshio region to the east of Taiwan and Japan.

**Fig 9:**
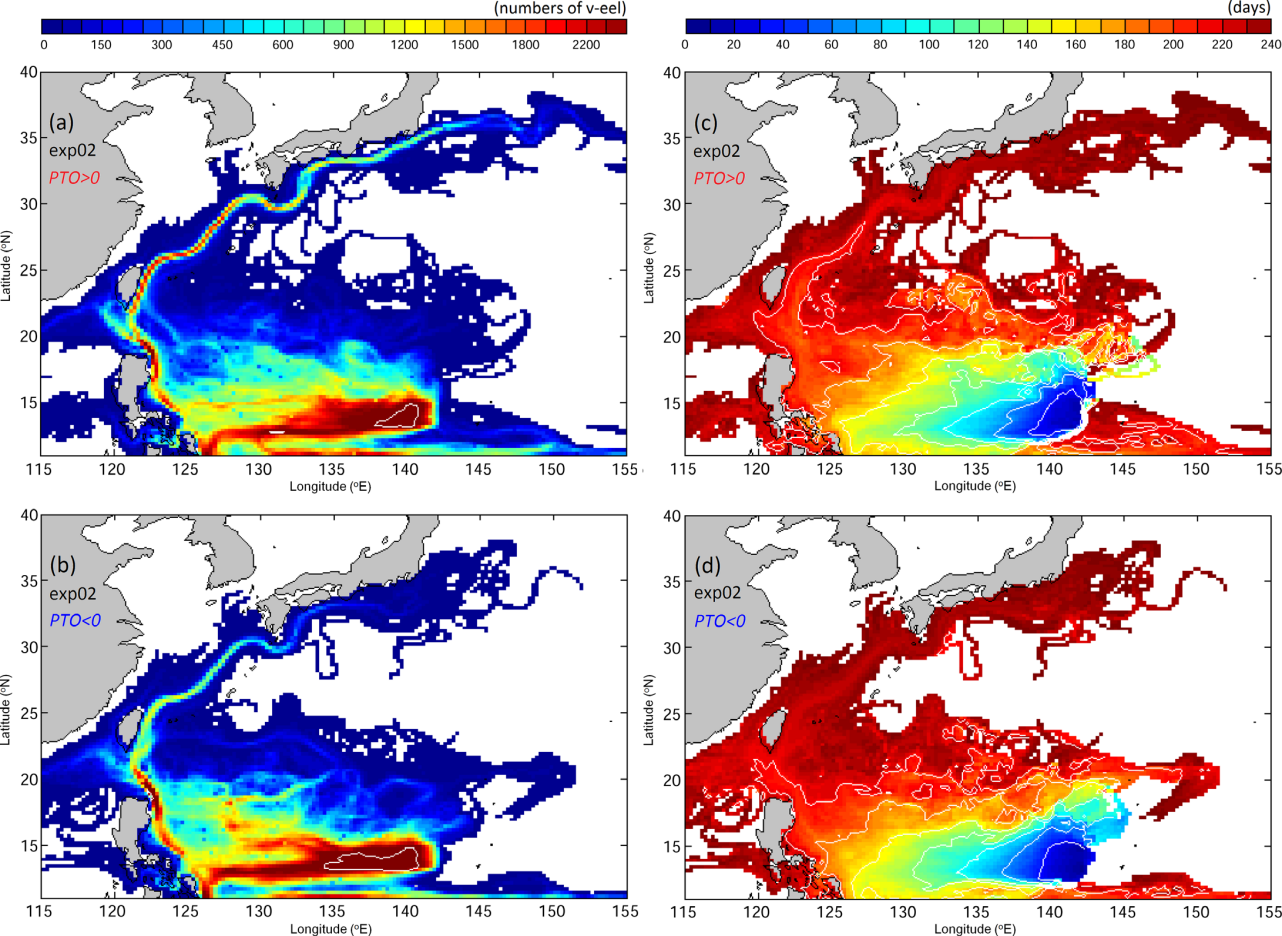
As for Fig 8, except that the v-eels have active swimming behavior (Exp02).

In previous numerical studies, the swimming ability of eels has often been neglected [6,34,35]. The VF distributions in Exp02, in which swimming ability is added to the v-eels from Eq 3, significantly differ from Exp01 results (Figs 8 and 9). A substantial number of v-eels in Exp02 reach the Kuroshio after seven months, and some arrive in the East China Sea in 7–8 months (early December to late February), in good agreement with observations (Figs 2 and 9). During the positive PTO phase, more v-eels arrive in the region south of Japan and v-eels in the NEC drift westward faster than in the negative PTO phase.

**Fig 10:**
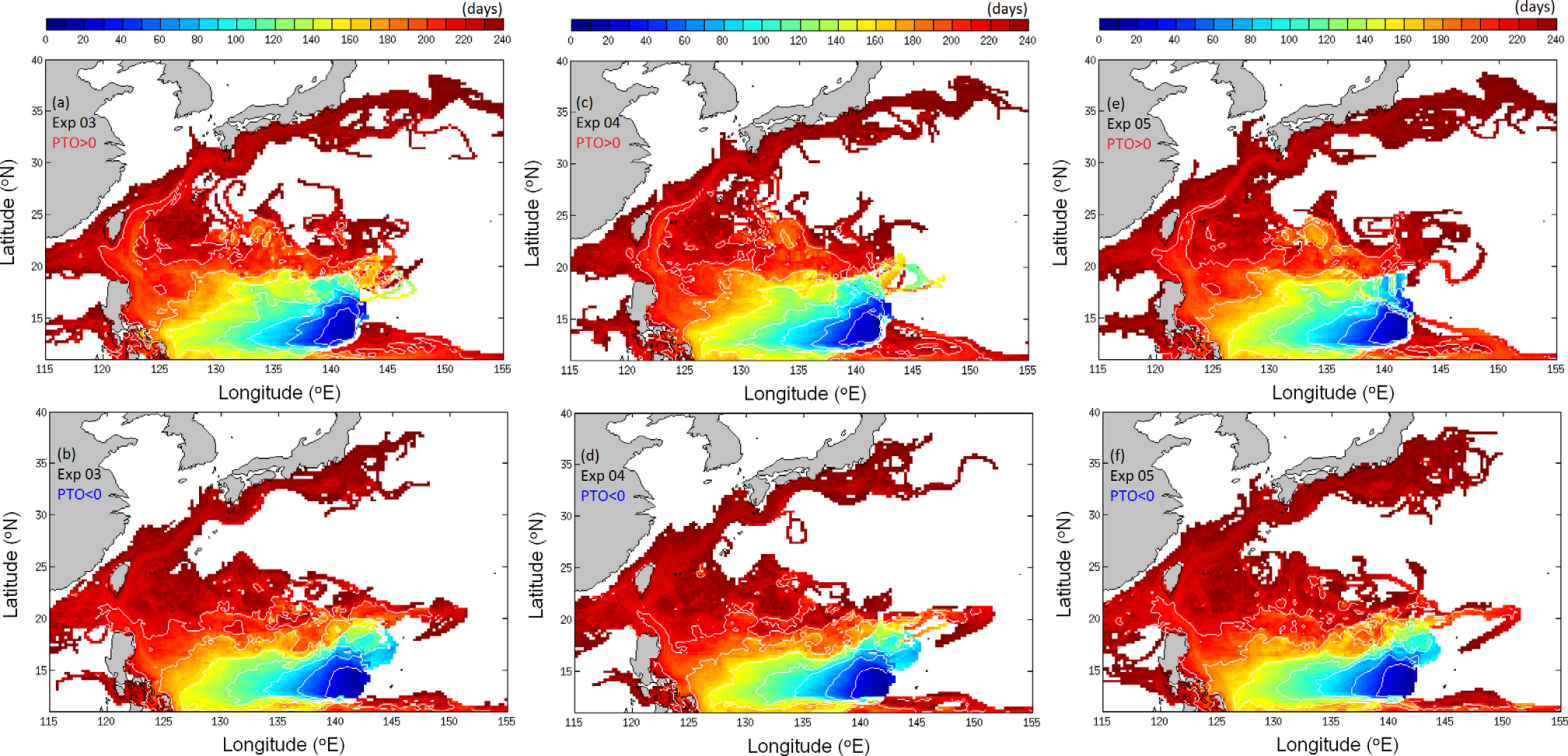
As for Fig 8, but for Exp03 (a,b), Exp04 (c,d), and Exp05 (e,f).

To confirm that the diversity of tracks shown in Exp02 under different climate regimes is robust, sensitivity experiments are conducted. As eel size does not increase linearly after metamorphosis into glass eels [38], Exp03 left the horizontal swimming speed unchanged after day 150. Exp04 tests the slower swimming speed with DVM unchanged from Exp02. Exp05 repeats the two-layer method used in former studies. The v-eels remain at 150 m during daytime and float to 50 m at night. The horizontal swimming speed in Eq 3 is again used. VFs for the three sensitivity experiments are similar to those of Exp02 (Fig 10). The results of Exp02 will be used as the standard in the following analysis.

We calculate the number of v-eels reaching the Kuroshio to the east of Taiwan and the area to the south of Japan (sections are marked in magenta lines in Fig 1) within eight months of tracking for all the experiments (Table 2). The probability of passively drifting v-eels reaching the Kuroshio is very small (<0.7%), and none reach the area south of Japan during the eight-month period. The experiments with swimming ability yield a significantly increased number of eels in the Kuroshio. In Exp02, 21% of v-eels arrive east of Taiwan and 8.1% arrive in the area south of Japan in positive PTO years. The numbers of v-eels reaching these two areas are reduced in the negative PTO period, with 14.4% of v-eels reaching the area east of Taiwan and 1.4% reaching the area south of Japan. The results of Exp03 and Exp05 are similar to those of Exp02. The smaller number in Exp04 is expected from the slower swimming speed. The differences between the two climate phases are significant (exceeds 5% significant level).

**Table 2:** Percentage of v-eels that appeared in the Kuroshio. The locations of transects are marked in Fig 1 (magenta lines).

Section	East of Taiwan		South of Japan	
PTO phase	Positive (+)	Negative (−)	Positive (+)	Negative (−)
Exp01	0.7	0.1	0	0
Exp02	21.0	14.4	8.1	1.4
Exp03	18.2	5.9	10.5	0.9
Exp04	16.7	11.3	4.9	0.8
Exp05	20.9	16.7	7.6	1.9

**Fig 11:**
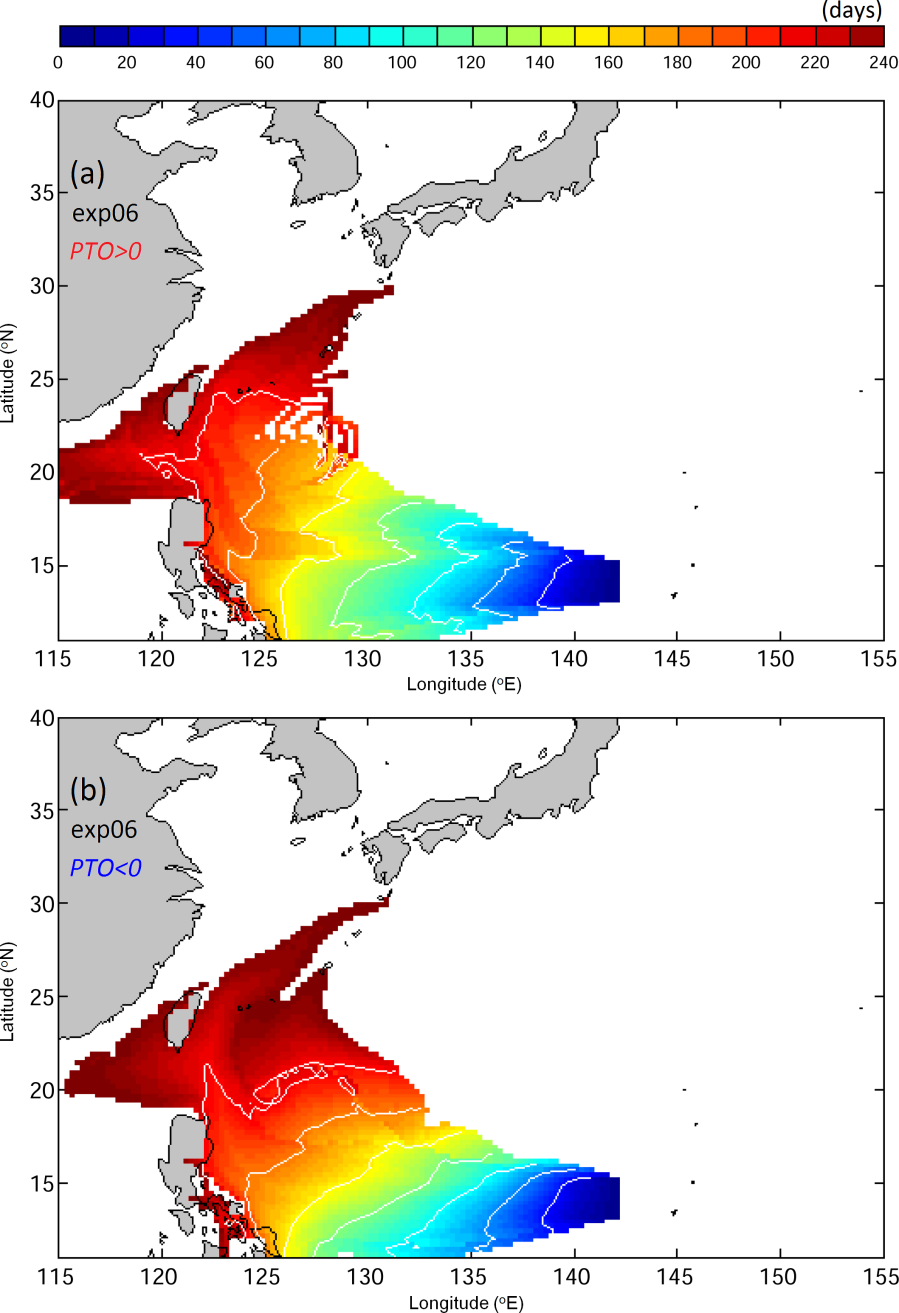
As for Fig 8, but using coarser ECMWF ocean currents in Exp06.

In Exp06, movements of v-eels are tracked using 3D currents from the ECMWF-ORAS4 reanalysis, which has a coarser resolution but cover a longer period than the JCOPE2. The VF distributions for Exp06 (Fig 11) reveal that use of coarse-resolution ocean currents causes rapid transport of v-eels by the NEC during the positive PTO years, but no v-eels in this experiment could reach the area south of Japan. This suggests that fine-resolution ocean currents are essential to generate more reliable movements of Japanese eels in the study region.

### Effect of environmental condition changes in two climate regimes

The effect of changes in physical oceanographic conditions on the migrations and distributions of v-eels is examined in this section. The final locations of v-eels are classified by region (Table 3). During the positive PTO phase, more v-eels arrive in the area south of Japan and the South China Sea and a larger number of v-eels continue in the NEC area, with fewer v-eels appearing in the East China Sea and STCC region compared to the negative PTO phase (Table 3).

**Table 3:** Final destinations of v-eels (percentages).

Regions	Positive (+)	Negative (−)
NEC	57.2	47.4
STCC	10.6	20.8*
South China Sea	4.8	4.1
East China Sea	5.0	6.6
South of Japan	8.6	3.4

*The number reduces to 13.7% if the spawning ground shifts southward

**Fig 12:**
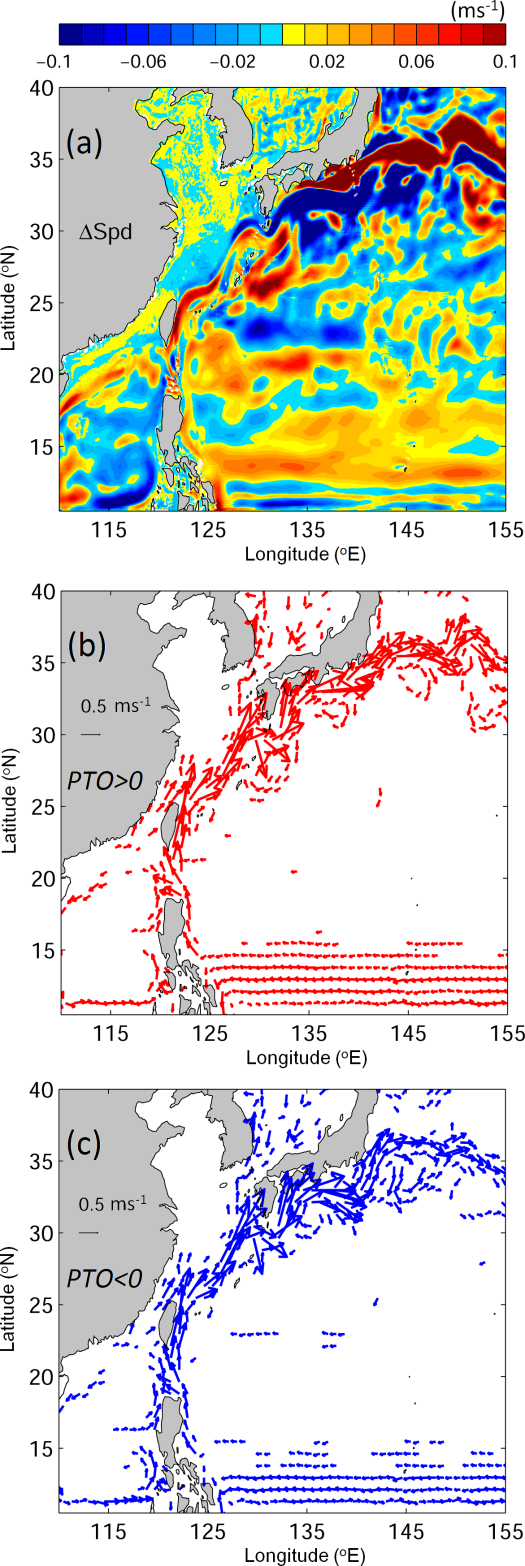
(a) Speed differences (ms^-1^) of vertically averaged currents in the top 300 m between the positive and negative PTO phases. Vertically averaged currents (ms^-1^) in the top 300 m for (b) the positive and (c) the negative PTO phase.

The large differences in the movements and distributions of v-eels between the two PTO phases can largely be explained by differences in the physical environmental conditions between the two phases (Fig 12). The Kuroshio is generally stronger during the positive PTO years because of stronger wind forcing, yet the strengthening is non-uniform because of modification by eddies (Fig 12; [39]). Once the v-eels enter the Kuroshio, they can be transported faster in the positive PTO phase than in the negative PTO phase (Fig 9). Differences in ocean currents also occur along the Kuroshio path between the two phases; during positive PTO years, the Kuroshio is weaker in the Luzon Strait than in the negative PTO years (Fig 12A). The Kuroshio weakening near the Luzon Strait is locally caused by the larger number of cold eddies [39]. The weakening of the Kuroshio produces a weaker potential vorticity jump across the jet, leading to the stronger Luzon Strait intrusion. As a result, more v-eels are able to reach the South China Sea (Fig 9 and Table 3) through the stronger Luzon Strait intrusion in the positive phase. The Kuroshio in the East China Sea shifts offshore in the positive PTO years (Fig 12A) because of forcing by wind stress curl and surface heat flux [40]. In addition to the fact that a stronger Kuroshio directs eels downstream to the south of Japan, the offshore swing of the Kuroshio may also play an important role in affecting the larval eel migration, causing fewer eels to reach the East China Sea in the positive PTO years than in the negative PTO years. Further downstream in the Kuroshio to the south of Japan, large changes in the current speed between the positive and negative PTO phases are mainly caused by the Kuroshio meanders (Fig 12). During the positive PTO years, the Kuroshio tends to follow the near-shore route over the region to the south of Japan (135–142°E, 31–35°N). The migration paths of v-eels in the positive PTO years in Exp02 reflect changes in physical oceanographic conditions, and are closer to the coast than in the negative PTO years (Fig 9).

**Fig 13:**
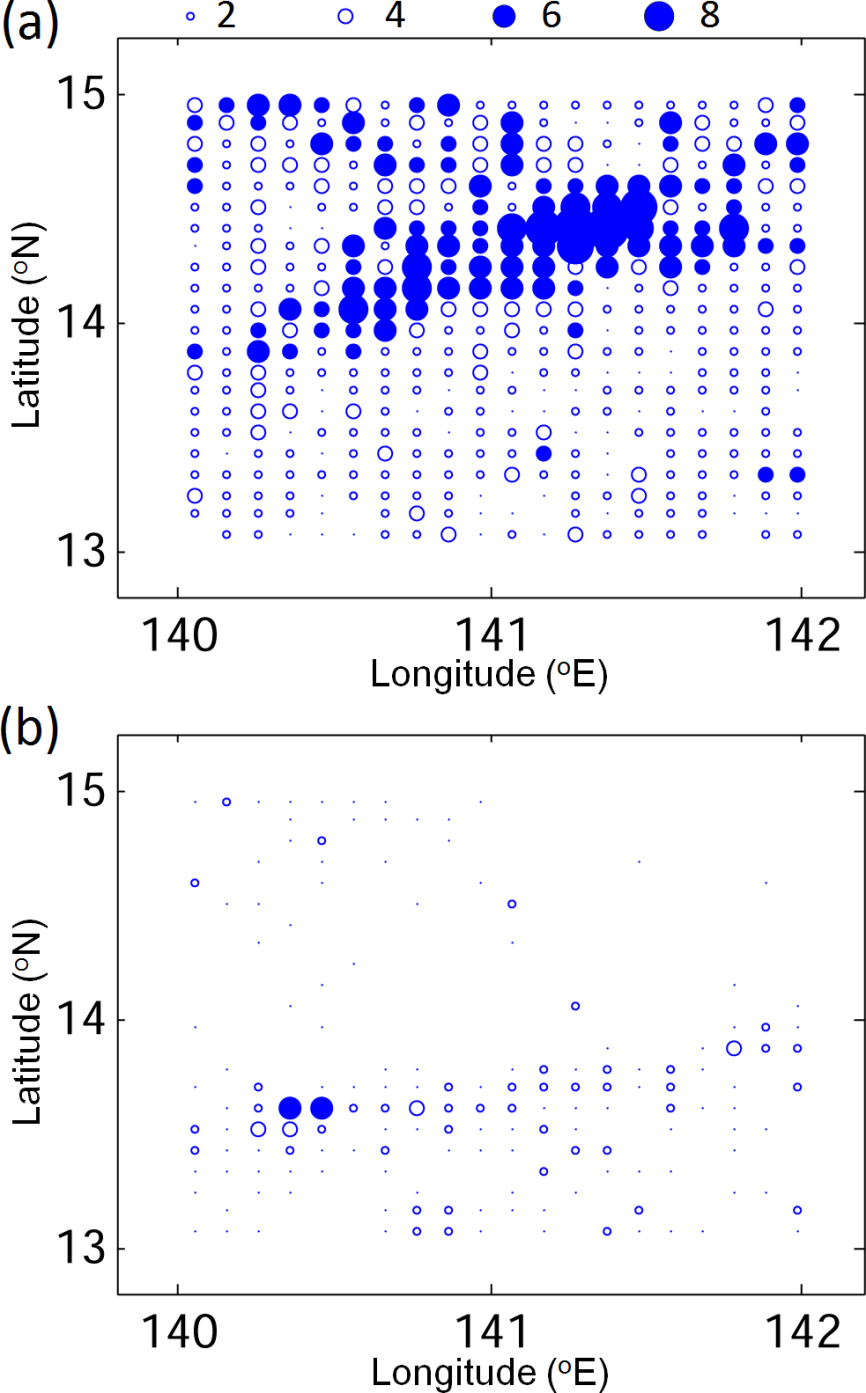
Origins of eels arriving south of Japan for (a) the positive PTO, and (b) the negative PTO. Shading indicates more than five eels.

More v-eels reach the area south of Japan in the positive PTO phase than in the negative phase. Other than the favorable condition provided by the Kuroshio, the eels may also be sensitive to the spawning locations, which are influenced strongly by the NEC. We extract the eels that arrive in the area south of Japan within eight months, and their origins for both climate phases are shown in Fig 13. During the positive PTO phase, v-eels from all the locations in the spawning ground can reach the area south of Japan. A large percentage of v-eels originate from the northern half of the spawning ground in the positive PTO phase (Fig 13A). The limited number of eels reaching the area south of Japan in the negative PTO phase mostly come from the southern part of the spawning ground (Fig 13B). During the positive PTO phase, the NEC bifurcation latitude shifts northward (Figs 3 and 12B; [23]). The spawning ground (between 13–15°N) is therefore located near the center of the NEC (Fig 12B). The eels in the northern half of the spawning grounds are directed to the Kuroshio system, while the eels in the south are likely to be transported to the Mindanao Current (Fig 12B). In contrast, the NEC moves southward in the negative PTO years (Figs 3 and 12C), and the northern spawning ground is no longer inside the NEC system as a result of this shift (Fig 12C). Only v-eels traveling from the southern spawning ground have a good chance of joining the Kuroshio and arriving in the area south of Japan.

The STCC region is known for its high eddy activity [41], and positive PTO years are characterized as eddy-rich years [23]. The JCOPE2 reanalysis captures well the main differences between the eddy-rich and eddy-poor years [39]. The STCC eddies are nonlinear [39]; this nonlinearity implies that fluid is trapped within the eddy interior [42]. Eel larvae may also be trapped by eddies. The increase in eddy activities is expected to have a large influence on v-eels; however, our results demonstrate that fewer v-eels remain in the STCC region in the positive PTO phase (eddy-rich years) than in the negative PTO phase (eddy-poor years). The number of v-eels remaining in the STCC region is associated with the bifurcation of the NEC. The northward position of the NEC in the positive PTO years covers almost the entire spawning ground, transporting a large amount of eels westward. During the negative PTO phase, the eels from the northern spawning ground cannot be transported by the NEC due to the southward shifting of the current, and thus drift into the STCC region.

To further examine the role of the NEC, we repeat the negative PTO experiment, but shift the spawning ground southward (12–14°N), so that the NEC covers a larger spawning area than the original case. Fewer eels remain in the STCC region (Table 3) and most travel downstream with the NEC.

### Calculation of otolith increment

**Fig 14:**
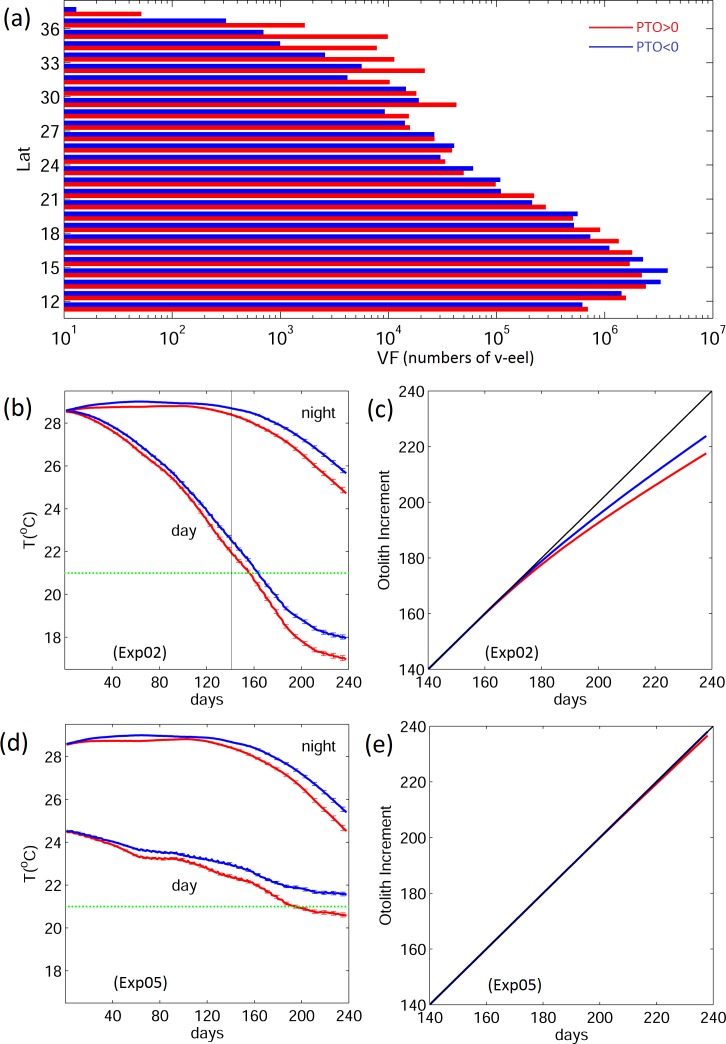
(a) VF latitudinal distribution; (b) and (d) temporal evolution of along-path water temperature; and (c) and (e) otolith increment for (middle) Exp02 and (bottom) Exp05. Red indicates the positive PTO, blue the negative PTO. The green line represents the temperature threshold (21°C) above and below which the increments are calculated differently (Eq 4).

The composite water temperature along the v-eels’ trajectories indicates that the eel larvae experience cooler temperatures in positive PTO years, particularly later than six months after release (Fig 14B). The temperature range is reflected in the v-eel distributions (Figs 9 and 14A). In latitudes higher than 29°N, the number of v-eels in the positive phase of PTO is much larger than in the negative PTO years (Fig 14A). The otolith increment for both climate phases is calculated according to Eq 4. The otolith increments are identical for both climate phases during nighttime and daytime initially (Fig 14C). After day 160, the daytime temperature drops below 21°C and the otolith increment starts to depart from the one-to-one line (Fig 14C). The otolith increment is less than the actual number of elapsed days, indicating that it underestimates ages in both climate phases. The cooler temperature in the positive PTO years results in larger underestimates.

Although the two-layer DVM (Exp05) results in a similar migration distribution to full 3D tracking (Exp02), the along-path temperature shows notable differences (Fig 14D). The temperature change with time is gentler in Exp05 due to the bounded lower layer at 150 m depth. The along-path temperature from Exp05 is cooler at the beginning of the simulation and becomes 3–4°C warmer than Exp02 towards the end of the simulation. The otolith increment in Exp05 is therefore different (Fig 14E), as the temperatures are above the threshold most of the time; nevertheless, differences between the two climate regimes still occur in Exp05.

## Discussions

This study examined the impact of circulation variability associated with PTO on the Japanese eel larval migration over wNP based on movement of virtual eels (v-eels) calculated using an individual-based model (IBM). IBM was driven by the 3D ocean circulation reanalysis produced by the JCOPE2. Two important biological behaviors, diel vertical migration and horizontal swimming ability, were included in IBM. It was demonstrated that the interannual circulation variability associated with the PTO significantly affects the eel larval migration path (and therefore their final distribution) and age estimates based on otolith increments.

**Fig 15:**
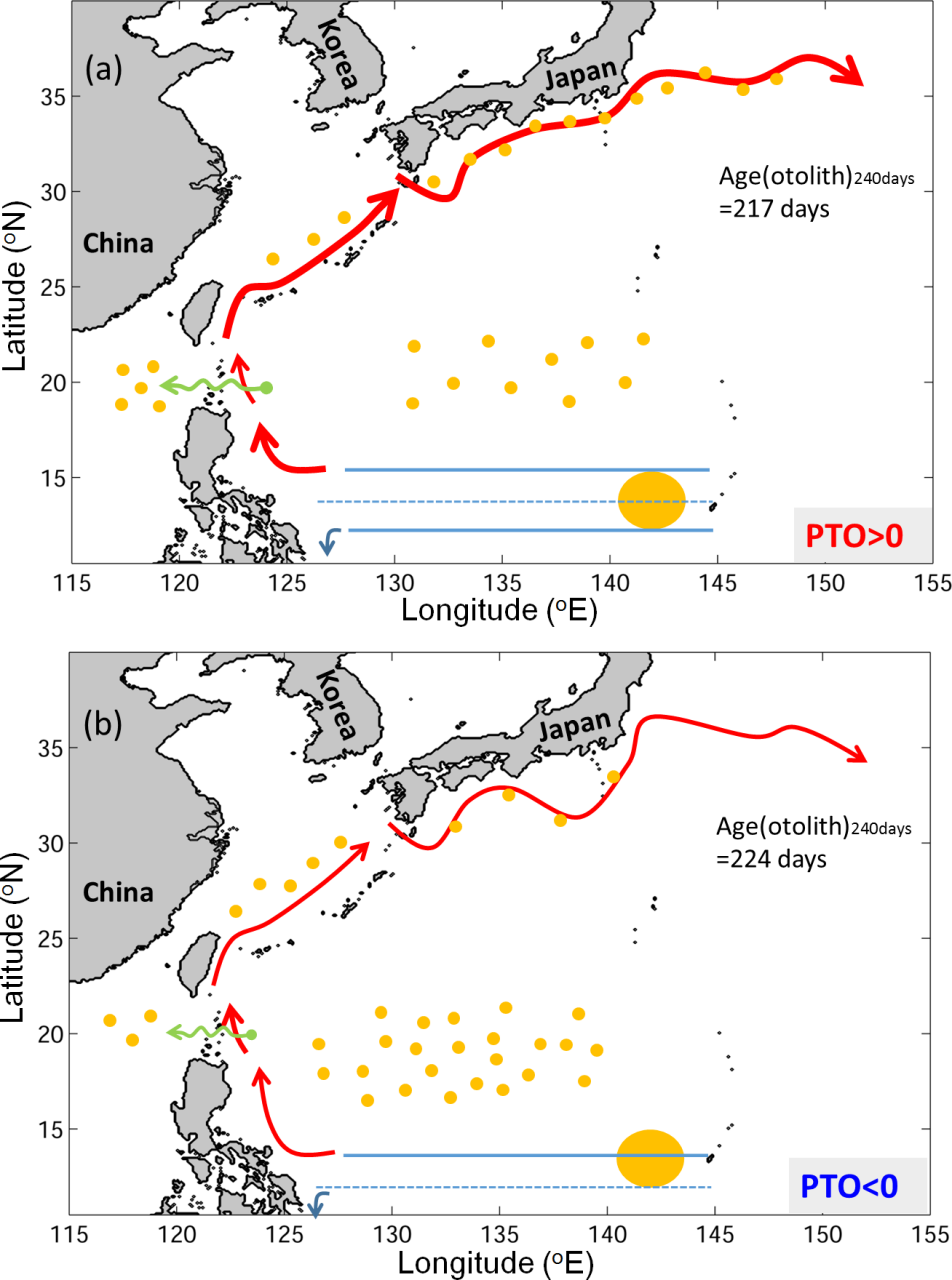
Schematic showing ocean conditions and v-eel distribution for (a) the positive PTO and (b) the negative PTO. The large yellow circle represents the spawning ground. Small yellow dots represent v-eels: the number of dots indicates the proportion reaching different areas (Table 3). Red curves represent the Kuroshio. Solid blue lines show the boundary of the NEC; the dashed line marks the center of the NEC. Green arrows represent intrusion through the Luzon Strait.

The main finding of this study is summarized in Fig 15. During the positive PTO years, the northward-shifted North Equatorial Current (NEC) covers the entire spawning ground. The v-eels are first transported westward from the release areas by the NEC. Some v-eels join the Kuroshio or drift into the STCC region; others enter the Mindanao Current. The wind-forced stronger Kuroshio in the positive PTO years brings the eels downstream, with more v-eels eventually reaching the area south of Japan than in the negative PTO phase. Due to local weakening of the Kuroshio in the Luzon Strait, more v-eels enter the South China Sea through intrusion. The offshore-shifted and faster Kuroshio in the East China Sea leads to fewer v-eel arrivals in the East China Seas in the positive PTO years than in the negative PTO years. The onshore position of Kuroshio to the south of Japan brings the v-eels close to the Japanese coast during the positive PTO years.

The differences in the total number of v-eels remaining over the STCC region between the two PTO phases mainly result from bifurcation of the NEC. In the positive PTO years, the northward shift of the NEC brings a large portion of v-eels westward toward the Kuroshio, with few v-eels remaining in the STCC area. During negative PTO years, due to the southward shift of the current, only eels from the southern spawning ground have a good chance of joining the NEC. A large amount of larvae from the northern spawning ground drift to the STCC region. Eddies in the STCC area may also play a role in carrying larvae toward the Kuroshio. The larger number of eddies in the positive PTO years in comparison with the smaller number of eddies in the negative PTO years may enhance the differences between the two climate regimes. The detailed interactions between eels and eddies are a topic for future studies.

El Nino was previously shown to possess a possible link to the Japanese eel recruitment [19,21]. The main physical processes for such a link, however, were not understood [18,22]. Using the PTO index, which directly influences the circulation over the western Pacific Ocean, this study demonstrated the significant impact of ocean environmental conditions on the Japanese eel migration and distribution over wNP. We noticed that the recruitment index of Japanese eel arrival to the coastal waters of Taiwan [22] correlates with the PTO index. The recruitment index is higher in the negative PTO years and lower in the positive PTO years. The reason may be that the faster and more offshore Kuroshio directs eels downstream and more eels enter the South China Sea, resulting in fewer eel arrivals to Taiwan during the positive PTO years. To interpret the conditions better, future research work should also include comparison of simulated v-eel distributions with observed eel catch data in East Asia in different PTO years. This study also demonstrated that active horizontal swimming behavior is necessary for larvae to escape the spawning area and reach the East Asian coastal waters. Active swimming behavior toward the fastest current was implemented in the numerical simulation of Bonhommeau et al. [43]. Rypina et al. [34] showed that active horizontal behavior in a random direction improved the success rates of larvae reaching the continental shelf, but directional swimming toward the continental shelf resulted in better success and a reasonable distribution along the North American shelf break. The means by which larval eels navigate or orientate remain unknown, but the behavior is necessary for eels to reach their growth habitat [34]; the present study]. Further field or laboratory experiments are required to understand the eels’ directional preference. The results should be used in further simulations to compare with the coastal distribution of larvae.

We demonstrated that a coarser resolution of 3D ocean currents leads to increased migration time, consistent with previous work [33]. The v-eels are transported more slowly in the coarser-resolution model and cannot reach the area south of Japan. This finding suggests that the fine-resolution ocean model is essential to obtain more precise results.

Calculation of the otolith increment suggests that eel age tends to be underestimated due to the cool temperatures experienced by larvae along the migratory paths regardless of the PTO phase. The underestimation in positive PTO years is more significant because of the eels’ distribution toward higher latitudes. Using the two-layer DVM, Zenimoto et al. [35] estimated the number of otolith increments of *A*. *japonica* to be one per day because the ambient water temperature is mostly warmer than 20°C. The two-layer DVM (Exp05) in the present work also leads to an absence of age underestimation because of the warmer temperature experienced by the v-eels. The age estimation is sensitive to the daytime diving depth due to the temperature distribution; conversely, the underestimation can be larger if eels dive deeper to cooler water. Further age estimates should take into account the diving depth and incorporate more observational information.

This study assessed the simulated Japanese eel larvae distribution for different climate phases in the western North Pacific Ocean by a 3D particle tracking method (with swimming ability included). The method can be applied to different species and to other ocean basins. Several issues remain unclear, such as the effect of eddies on larvae and how larvae pass across the strong Kuroshio and reach the continental waters. Natural mortality may also influence the final distribution of eel larvae, which is another topic for future studies.
